# Bayesian Sensitivity Analysis of a Cardiac Cell Model Using a Gaussian Process Emulator

**DOI:** 10.1371/journal.pone.0130252

**Published:** 2015-06-26

**Authors:** Eugene T Y Chang, Mark Strong, Richard H Clayton

**Affiliations:** 1 Insigneo Institute for *in-silico* Medicine, University of Sheffield, Sheffield, United Kingdom; 2 Department of Computer Science University of Sheffield, Sheffield, United Kingdom; 3 School of Health and Related Research, University of Sheffield, Sheffield, United Kingdom; Oxford University, UNITED KINGDOM

## Abstract

Models of electrical activity in cardiac cells have become important research tools as they can provide a quantitative description of detailed and integrative physiology. However, cardiac cell models have many parameters, and how uncertainties in these parameters affect the model output is difficult to assess without undertaking large numbers of model runs. In this study we show that a surrogate statistical model of a cardiac cell model (the Luo-Rudy 1991 model) can be built using Gaussian process (GP) emulators. Using this approach we examined how eight outputs describing the action potential shape and action potential duration restitution depend on six inputs, which we selected to be the maximum conductances in the Luo-Rudy 1991 model. We found that the GP emulators could be fitted to a small number of model runs, and behaved as would be expected based on the underlying physiology that the model represents. We have shown that an emulator approach is a powerful tool for uncertainty and sensitivity analysis in cardiac cell models.

## Introduction

The heart is an electromechanical pump. With each beat an electrical action potential originates in the natural pacemaker, and propagates through the entire organ, acting to initiate and synchronise contraction. Since the publication of the first model describing the cardiac action potential over 50 years ago [1], models of electrical activation and recovery in cardiac cells and tissue have become valuable research tools. These models have explanatory power as they express quantitatively our knowledge of the biophysical processes that generate the cardiac action potential. Thus they can be used to examine the mechanisms by which gene mutations, pharmaceuticals and diseases act to change the properties of electrical activity in single cells and in whole tissues [2–4]. The present generation of models include detailed descriptions of the dynamics of transmembrane current flowing through ion channels, pumps and exchangers in the cell membrane, coupled to detailed models of Ca^2+^ storage and release within the cell [4], and there has been an increasing trend from models of animal cells towards detailed models of human atrial [5] and ventricular [6, 7] myocytes. These models can be coupled to models of cardiac tissue and anatomy [8], and there is the prospect that they will be used not only to interpret and guide experimental work [2], but also as tools to guide interventions in the clinic [9].

### Cardiac cell model parameters

Models of the cardiac action potential represent beliefs about current flow across the cell membrane. The dependence of these currents on voltage, time, and other quantities is controlled by parameters in the model, which are initially unknown. In the very first model of this type representing the squid giant axon [10], data from experiments informed both the functional forms of the model and parameter values for just three ion channel currents. The present generation of cardiac cell models represent current flow through many different ion channels, pumps, and exchangers, and are typically a set of stiff and non-linear ordinary differential equations with many parameters (for example, see [7]).

Parameter values for these equations are chosen based on experiments on populations of cells, usually conducted in different institutions, on different species, and under different conditions [11]. A further complication is that experimental action potentials produced by a population of cells have a range of shapes [12, 13]. Part of this variability can be attributed to differences in cell size, in the density of ion channels, pumps, and exchangers in the cell membrane, and in stochastic ion channel gating that can result in beat to beat differences in action potentials recorded from the same cell [13–15]. The uncertainty that accompanies model parameter values that are estimated from experimental data arises then not only from experimental error, but also the inherent variability in action potentials. [7, 16].

### Parameter sensitivity and uncertainty

Parameter values in this type of model will never be known with certainty, and so it is important to characterise the effect of this uncertainty on model outputs, and on conclusions drawn from modelling. Sensitivity analysis is the analysis of the influence of model parameters on model behaviour and is important in the context of cardiac cell modelling for three main reasons. First, parameters are estimated from uncertain experimental data. Understanding how the uncertainty in parameter values results in uncertainty in the conclusions drawn from a model-based analysis is important, for example in understanding how pharmacological agents or gene mutations influence the action potential. Second, we may wish to prioritise learning about a particular parameter, or set of parameters, if by doing so we will improve the soundness of our conclusions. Conversely, we may be willing to fix a parameter if it has negligible effect on the model output, and thus reduce the number of parameters that require estimation. Last, in the stiff, non-linear systems that typify cardiac cell models, small changes in parameter values may result in markedly changed model behaviour. These effects may be critical when we are seeking to explore and understand the effects of drugs, gene mutations, or regional differences in ion channel expression.

Several studies have addressed the issue of parameter sensitivity through running large numbers of simulations, each with a different combination of parameters [12, 17, 18]. However this approach is challenging because, despite the relentless increase in computer power, large numbers of model runs can still be computationally demanding. Other studies have taken a different approach, where outputs from a cardiac cell model are used to build a statistical ‘emulator’ or ‘meta-model’, which can then be used to calculate parameter sensitivity [19, 20]. For example, Sobie et al [19] used a partial least squares (PLS) linear model as an emulator for the cardiac cell model. Outputs of interest (for example, the action potential duration) were expressed as weighted sums of model parameters, where weights were derived from PLS regressions of simulator output quantities on simulator inputs in a set of training data.

### Gaussian process emulators

In this paper we describe an approach to emulation based on modelling the cardiac cell model using a *Gaussian process*. Gaussian process (GP) emulation is the dominant approach for the statistical modelling of computer model functions [21]. GPs have proven to be flexible in fitting a wide range of computer model functions, and they allow the straightforward assessment of both uncertainty (what is our uncertainty about the output quantities given our uncertainty about the input parameters?) and input sensitivity (what effect does changing an input have on the output?) [22, 23].

In keeping with the computational model uncertainty literature, we describe the cardiac cell model as a *simulator*. The simulator, can be thought of as a function *f*_*s*_(⋅) that generates a vector of outputs **y** given a vector of inputs (we use ‘inputs’ interchangeably with ‘parameters’ from now on), **y** = *f*_*s*_(**x**). The ‘true’ or ‘correct’ values of the input parameters **x** are typically unknown and we represent our knowledge about the values via the probability density function *p*(**x**). For the purposes of this paper we assume that our simulator is deterministic, i.e. that if we run the simulator repeatedly with the same set of inputs **x**′ we will in each case observe the same model output value **y**′.

Our *emulator* is a statistical model of the simulator, sometimes known as a meta-model, a surrogate model, or a response surface model. The emulator is a probability distribution for the function *f*_*s*_(⋅). Given any point in parameter space **x** the emulator encodes our knowledge about the corresponding model output **y**. If we have run the simulator at **x**, then we know the corresponding model output with certainty. Note that this does not mean that we know with certainty the ‘true’ value of the quantity we are modelling, just that we know what value the computer model will return given input parameters **x**. However, for values x* for which we have not run the model, we are uncertain about y* = *f*_*s*_(x*). We therefore use our emulator to derive the expectation and variance of y*, and indeed any other distributional quantity of interest. Our emulator is typically cheap to compute compared to the simulator, and we can therefore use it to predict **y** for choices of inputs **x** over a wide range of parameter space. We can also use the emulator to explore the sensitivity of the model output to changes in the model input parameters, and it is here that the GP emulation method is particularly computationally efficient [22, 23].

The methodology for the GP emulation approach is described in detail in the ‘MUCM toolkit’, developed as part of a project on Managing Uncertainty in Complex Models (http://mucm.aston.ac.uk/MUCM/MUCMToolkit/). Good examples of the application of the method are to examine complex computer models of galaxy formation [24] and atmospheric chemistry [25].

### Aim of the study

Our aim was to build GP emulators for the outputs of a simple cardiac cell model, and to use these emulators to examine how uncertainties in maximum channel conductances translate into uncertainties in outputs describing action potential shape and restitution. We deliberately chose the simplified cardiac cell model developed by Luo and Rudy in 1991 (LR1991) [26], where the relationship between parameters and model outputs is relatively well characterised, so that we could examine the utility of an emulator approach and make comparisons with previous work on sensitivity analysis [19].

## Materials and Methods

### Implementation of cardiac cell model and choice of inputs and outputs

The LR1991 model (the simulator) was implemented in Matlab (Mathworks, CA) using code automatically generated from the CellML repository (www.cellml.org), which uses the Matlab ode15s time adaptive solver for stiff systems of ODEs. We chose to vary the six model input parameters representing maximum conductances. This was in line with a previous sensitivity analysis of the LR1991 model that used PLS regression [19]. These parameters have a clear link to cellular physiology as they represent the density of ion channels (or pumps and exchangers) in the cell membrane. Furthermore, the contribution of each of the currents in the LR1991 model to the action potential is understood, at least qualitatively [4, 26].

We were interested in eight model outputs. Six of these were obtained directly from the action potential: maximum dV_m_/dt, peak V_m_, dome V_m_, action potential duration to 90% repolarisation (APD_90_), resting V_m_, and APD to 50% of repolarisation (APD_50_). These outputs correspond to the biomarkers used in the study of Britton et al [12], except that we substituted APD_50_ for plateau duration because we found this could be measured more consistently. In addition to these six outputs, we also measured the maximum slope of the action potential duration restitution (APDr) curve and the minimum diastolic interval (DI) of the shortest S2 stimulus that could elicit an action potential.

### Design data and test data for the emulator

To build a GP emulator it is necessary to generate a design dataset, which consists of a set of parameter inputs and the corresponding simulator outputs. The range of inputs should provide good coverage of the input parameter space, so that the emulator can represent the model over this plausible space. The design data are used to build, or ‘fit’, the emulator, and once the emulator is fitted, it can then be evaluated against a test dataset, which are obtained from simulator runs that have not been used in the fitting process.

In the original description of the LR1991 model [26], values for the maximum conductances were chosen based on earlier models and on experimental data. Our aim was to use the emulator to undertake sensitivity analysis over a plausible region of model parameter space, rather than to chose inputs that represented beliefs (in a probabilistic sense) about the ‘true’ maximum conductances. For the design and test data, we therefore selected combinations of maximum conductances using Latin hypercube sampling [27] from a uniform distribution in the range $\bar{G_{x}}\pm \bar{G_{x}}/4$ where $\bar{G_{x}}$ was the maximum conductance provided in the original description of the LR1991 model. This range of parameters was chosen to avoid inputs (for example G_K_ <0.2 mScm^-2^) that would generate prolonged repolarisation in the LR1991 model, and is shown in Table 1. The uniform distribution of each input was normalised to the range [0, 1] with the original conductance reported for the LR1991 model corresponding to 0.5. Thus normalised G_Na_ varied from 0.0 corresponding to 17.25 mScm^-2^ on the natural scale, to 1.0 corresponding to 28.75 mScm^-2^. For uncertainty and sensitivity analysis using the emulator, the inputs were assigned a Normal distribution with a mean of 0.5 and a variance of 0.04 (standard deviation 0.2) in normalised units. These values were chosen so as not to stray outside the range of the design data used to construct the emulators, and the mean and variance of each input in natural units is also given in Table 1.

**Table 1:** Distribution for input parameters.

Input parameter	Units	Mean value	Distribution for design data	Distribution for sensitivity analysis
G_Na_	mS cm^-2^	23.0000	U(17.250, 28.750)	N(23.0000, 0.84640000)
G_si_	mS cm^-2^	0.09000	U(0.0675, 0.1125)	N(0.09000, 0.00001296)
G_K_	mS cm^-2^	0.28200	U(0.2115, 0.3525)	N(0.28200, 0.00012544)
G_K1_	mS cm^-2^	0.60470	U(0.4535, 0.7559)	N(0.60470, 0.00058564)
G_Kp_	mS cm^-2^	0.01830	U(0.0137, 0.0229)	N(0.01830, 0.00000064)
G_b_	mS cm^-2^	0.03921	U(0.0294, 0.0490)	N(0.03921, 0.00000256)

Distribution for each input to the LR1991 simulator used for generating the design data and test data, and the distribution of each input used for undertaking sensitivity analysis in the emulator. U denotes a uniform distribution and N a normal distribution, with mean and variance given in brackets.

We generated 200 design points with which to build emulators for each of the eight outputs. Each of the 200 design points represented a single evaluation of the model, i.e. a single sample of the six inputs, and a single set of the eight output quantities. These data are provided as Supporting Information (S1 Data). An additional 20 sets of inputs and outputs were generated for the purpose of testing the emulator, and these data are also provided as Supporting Information (S2 Data). The choice of 200 for the number of design points was obtained empirically; fitting the emulators with design data based on <150 design points resulted in a relatively poor fit when evaluated against test data, whereas building an emulator with >250 design points resulted in a very similar fit to that obtained with 200 design points.

**Fig 1:**
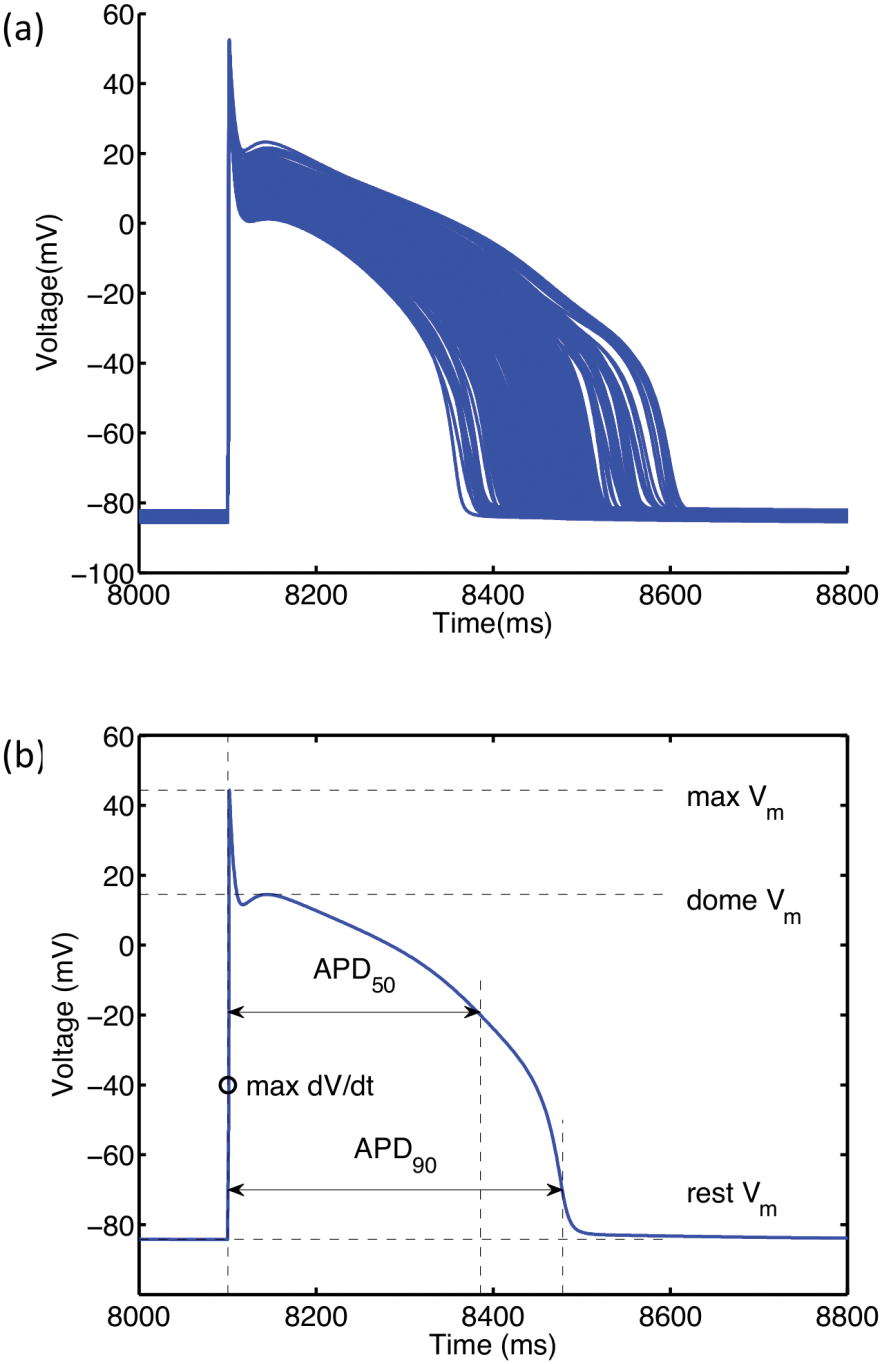
Outputs produced by the LR91 model. (a) Action potential biomarkers used as model outputs to characterise the model. (b) Action potential time series from 200 runs of the LR1991 model used as design data for the GP emulator.

Each time the simulator was run (for generating both design and test data) nine S1 stimuli of strength -25.5 μA cm^-2^ and duration 2 ms were delivered to the simulator at a 1000 ms cycle length. The final action potential in this S1 sequence was used to obtain the outputs. Fig 1(a) shows the 200 action potentials that were used to generate the outputs. Fig 1(b) shows the six outputs that were measured directly from the action potential.

The slope of the APDr curve and minimum DI characterise dynamic properties of the simulator and were obtained as follows. Following the initial simulator run to obtain the outputs shown in Fig 1(b), the simulator was run repeatedly using the final S1 beat of the initial run as an initial condition, with an S2 stimulus delivered at progressively shorter cycle lengths until an S2 beat could not be elicited. The stimulus strength and duration were -25.5 μA cm_-2_ and 2 ms as described above. From each of these additional runs, APD_90_ and diastolic interval (DI) were obtained from the S2 action potential, and used to construct an APDr curve. The minimum DI was the shortest DI in the S2 sequence. The maximum slope was determined by fitting an equation of the form

$$\begin{array}{c} APD=a-be^{-DI/c} \end{array}$$ (1)

to the APDr curve using the Matlab fminsearch function, where a, b and c were obtained from the fit. The maximum slope of the curve was then determined [28] from

$$\begin{array}{c} MaxSlope=\frac{a}{b}e^{-DI_{min}/b}. \end{array}$$ (2)

**Fig 2:**
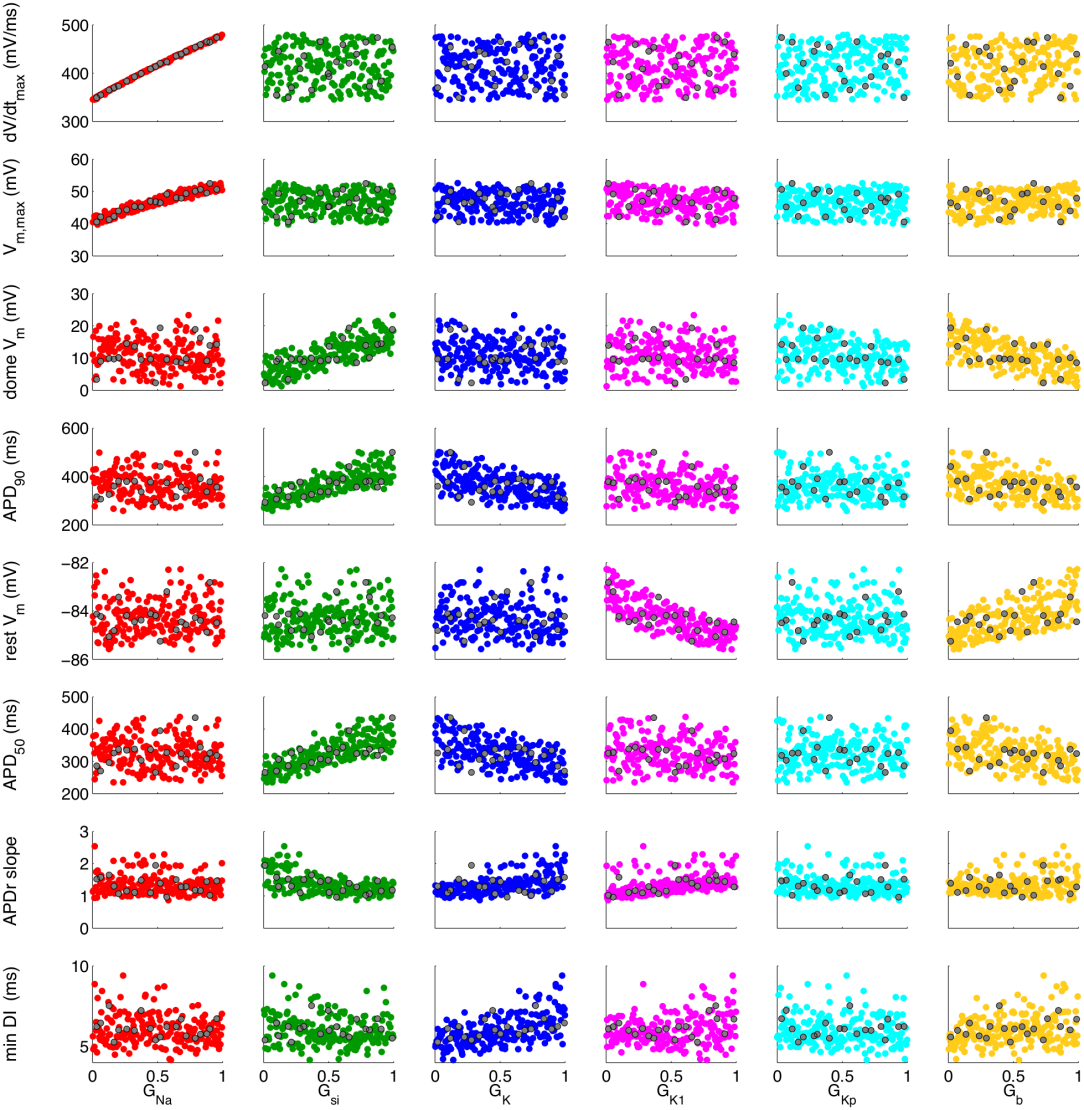
Design data and test data obtained from 220 runs of the LR1991 model for eight outputs and six inputs. Each plot shows combinations of inputs and outputs, with 200 coloured points indicating design data used to fit the emulators, and 20 grey points showing test data used to validate the emulator.

The design data inputs and outputs are plotted in Fig 2, where each plot shows one of the eight output quantities plotted against one of the six input quantities. Test data are overlaid as dark grey points. Several associations are immediately clear from this figure, for example maximum dV_m_/dT and peak V_m_ are both strongly associated with G_Na_ and weakly associated, if at all, with the other inputs. On the other hand, APD_90_ shows shows some dependence on G_si_, G_K_, and G_b_. Note that the inputs have been normalised such that they to lie in the (0,1) interval. The uniform sampling distribution for the inputs is clear from the even spread of points in the (0,1) interval.

### Building and evaluating the emulator

Our approach to Gaussian process emulator construction was based on that described in previous studies [23, 25], and a detailed description of the process and the underlying mathematics is given in the Supporting Information (S1 Text). We fitted a separate GP emulator for each of the eight outputs using the 200 sets of design data. Emulator fitting refers to the process of estimating emulator hyperparameters. In our study each emulator was characterised by a set of seven ‘mean function’ hyperparameters (an intercept $\hat{\beta_{0}}$, and six slope hyperparameters $\hat{\beta_{1}},...,\hat{\beta_{6}}$ corresponding to the inputs), and a set of seven ‘covariance function’ hyperparameters (six correlation lengths $\hat{\delta_{1}},...,\hat{\delta_{6}}$ controlling the ‘wiggliness’ of the emulator, and an overall variance hyperparameter ${\hat{\sigma }}^{2}$). Given design data and hyperparameter values, an emulator can be evaluated for any new set of inputs. An emulator evaluation for a new input is a Gaussian probability distribution with a defined mean and variance that represents our knowledge about the output of the simulator at that input.

The quality of the emulator fit was evaluated using the test data. The output distributions predicted by the emulator for the new inputs were compared with the actual outputs obtained from the simulator at the test points. A visual indication of fit was obtained by plotting the differences between the emulator means and the simulator outputs for each of the 20 test data points, and a quantitative indication was obtained by combining these differences into a single measure, the Mahanalobis distance (MD). This measure provides a robust way to compare the output of the emulator and the output of the simulator at the test points, expressed as a single number [29]. A reference distribution for the MD can be calculated, which for 20 points has a mean of 20 and a standard deviation of 6.8 [29]. A good quality emulator fit will predict the output at the test points correctly, and the MD will be close to the mean of the reference distribution.

When a satisfactory emulator fit had been obtained, the test data were then combined with the original design data and used to fit an updated emulator based on 220 simulator runs.

### Uncertainty analysis

Uncertainty analysis aims to quantify how uncertain we are about the target output quantity, given our uncertainty about the model inputs. The traditional approach to this problem is Monte Carlo analysis, whereby a large number of samples is generated from the input parameter distribution, and for each sample the model is run. The resulting set of model outputs represents a sample from a distribution that characterises the uncertainty about the output quantity. In contrast, an emulator can be used to specify directly an analytic distribution (in our case, a Gaussian process) that represents uncertainty about the output, given uncertainty in the inputs [30].

### Sensitivity analysis

For the purposes of our study, we assumed that uncertain inputs are normally distributed, and under this assumption we can derive analytically a range of sensitivity analysis measures that quantify the effect of individual inputs on each output (see S1 Text for details). In particular, we can determine the inputs that have the greatest (and least) influence on an output, thereby giving insight into the operation of our model. Without an emulator, these sensitivity measures would typically require a costly Monte Carlo procedure.

The emulator was used to assess the contribution of each input with respect to each output in *mean effect* plots (a plot of the expectation of the output quantity, conditional on the input of interest, plotted against the input of interest), and to calculate the *main effect index*, for each input-output combination [23, 30]. Sensitivity measures computed using the Gaussian process emulator were then compared with those computed using a PLS emulator [19] constructed using the same design data. The mathematical details of these procedures are described in detail in the Supporting Information (S1 Text), and are summarised here.

The *mean effect* of an input of interest, x_w_, on some output is the conditional expectation of that output, conditional on the input of interest, i.e. after averaging over the remaining inputs. This is a function of the given input x_w_, and enables the effect of varying a single input on the output to be examined. In each emulator, the mean effect of each of the six inputs was calculated in turn over the range 0 → *x*_*w*_ → 1, with all other inputs independently normally distributed with mean 0.5 and variance 0.04. A variance of 0.04 was chosen so that input values had good coverage over the (0,1) interval.

Sensitivity indices are a measure of the overall sensitivity of outputs to inputs, and can be calculated in several different ways. We calculated the *main effect index*, which for a parameter x_w_ is the ratio of the variance of the mean effect *V*_*w*_ = *Var*[*E*{*f*(**x**)∣*x*_*w*_}], to the variance of the model output *Var*{*f*(**x**}. If, by the variance identity, we write *Var*{*f*(**x**} = *Var*[*E*{*f*(**x**)∣*x*_*w*_}]+*E*[*Var*{*f*(**x**)∣*x*_*w*_}], we can see that the main effect index can be interpreted as the expected reduction in variance in the output that would occur if we were to learn (or fix) x_w_.

Again, because we used an emulator to calculate the sensitivity measure we took expectations with respect to the emulator, and calculated the ratio of the emulator expectation of the mean effect variance E*[*V*_*w*_] to the emulator expectation of the variance of the emulator output E*[*Var*[*f*(**x**)]]. The main effect index does not take into account any variance in the output that could be attributed to interactions between the inputs. However, in the absence of interactions the sum of the sensitivity indices for each input will be 1.0, and deviation from this is therefore a measure of the contribution of interactions.

The PLS emulator was generated following Sobie [19] using the NIPALS algorithm [31]. The combined design and test data used to fit the GP emulator were used to generate the PLS emulator. Inputs were first mean centred and normalised by the standard deviation to obtain Z scores. The Z scores of the input X, and the columns in output Y corresponding to APD_90_ and maximum dV_m_/dT were then log-transformed. The eight outputs were then regressed on the six inputs resulting in 8 × 6 regression coefficients.

## Results

### APD_90_ emulator

To explain the way that GP emulators were used in this study, we concentrate initially on the APD_90_ emulator, before presenting our findings for the other emulators.

**Fig 3:**
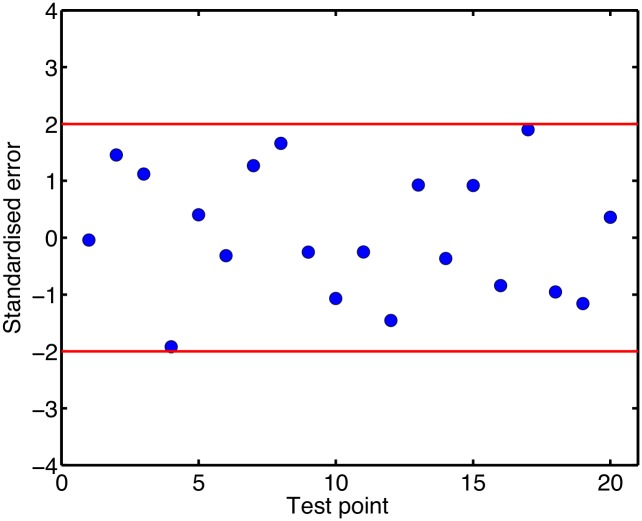
Validation of APD_90_ emulator output against test data output. This plot shows the difference in the mean APD_90_ predicted by the emulator and APD_90_ obtained from the simulator for each of the 20 test data. The difference is calibrated as the number of standard deviations, and the red lines indicate ±2 standard deviations.

The evaluation of the APD_90_ emulator against test data is shown in Fig 3, which shows the difference between the output of the emulator and the output of the simulator for each of the 20 test data. These differences all fall within ± 2 emulator standard deviations, indicating that the emulator is a good fit. The Mahalanobis Distance (MD) for these test data was 28.22, which is within the plausible range given the reference distribution (mean 20 and standard deviation 6.8) [29]. See the Supporting Information (S1 Text) for details of how the MD was calculated. We therefore concluded that the APD_90_ emulator was a good representation of the simulator.

**Fig 4:**
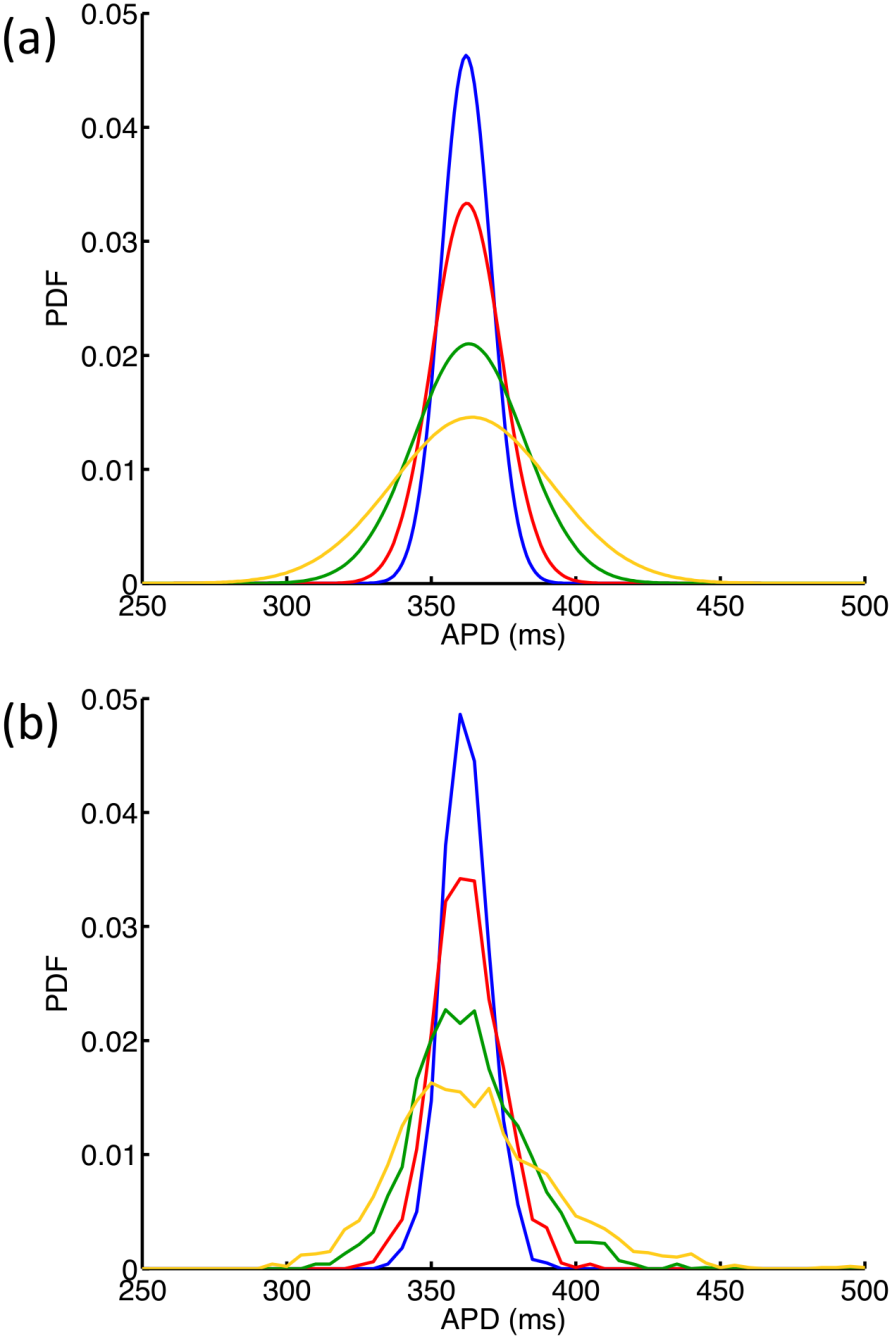
Variance in APD_90_ emulator. (a) Distributions of APD_90_ resulting from normal distributions of G_K_ when all other maximum conductances were effectively held constant by assigning a mean of 0.5 and a very small variance of 0.0001 in normalised units. G_K_ was assigned a mean value of 0.282 mS cm^-2^ and variance 0.0014 (blue) 0.0028 (red), 0.0071 (green), 0.0141 (yellow) mS cm^-2^. (b) Distributions of APD_90_ obtained from four Monte Carlo analyses, each with 2000 simulator runs, and with G_K_ drawn from distributions with mean value of 0.282 mS cm^-2^ and variance 0.0014 (blue) 0.0028 (red), 0.0071 (green), 0.0141 (yellow) mS cm^-2^.

We next assessed how the variance of APD_90_ calculated directly using the emulator depended on the variance of G_K_. For this calculation, all inputs except G_K_ were effectively fixed by assigning independent normal distributions with a mean in normalised units of 0.5 and a very small variance of 0.0001 in normalised units. G_K_ was then assigned a normal distribution with mean 0.5 in normalised units (0.282 mS cm^-2^ on the natural scale), but to show the effect of increasing uncertainty in G_K_, the variance was set at 0.01, 0.02, 0.05 and 0.1 in successive calculations of the mean and variance of APD_90_. These normalised variances correspond to standard deviations of 0.0014, 0.0028, 0.0071, and 0.0141 mS cm^-2^ on the natural scale. The output distributions of APD_90_ for each distribution of G_K_ are shown in Fig 4(a). As would be expected, increasing the variance of G_K_ results in an increase in the variance of APD_90_.

We then compared the output distributions of APD_90_ obtained from the emulator with output distributions obtained from a broadly equivalent Monte Carlo analysis of the simulator. Kernel density estimates were obtained using values from 2000 simulator runs, where in each run the input G_K_ was drawn from a normal distribution with the same variance as used to calculate the corresponding output distribution with the GP emulator. The number of runs was selected empirically as a value that yielded a smooth distribution of APD_90_ without incurring a large computational cost. The results are shown in Fig 4(b). The quantitative agreement between the distributions shown in Fig 4(a) and 4(b) confirms that the both approaches yielded a similar uncertainty analysis, and captured the behaviour of APD_90_ dependence on G_K_ in the LR1991 model. However, the GP emulator method required many fewer simulator runs and was therefore more efficient. The emulator approach (Fig 4(a)) required 220 simulator runs to generate design and test data, and once the emulator had been built (which took several minutes), the calculation of each new output distribution took only a few seconds. In contrast, each distribution of the Monte Carlo based approach (Fig 4(b)) was calculated from 2000 simulator runs (an order of magnitude more). While this study did not undertake a full benchmark comparison, we noted that computation times for each Monte Carlo run of 2000 samples varied from 9 hours 13 min to 9 hours 34 min.

**Fig 5:**
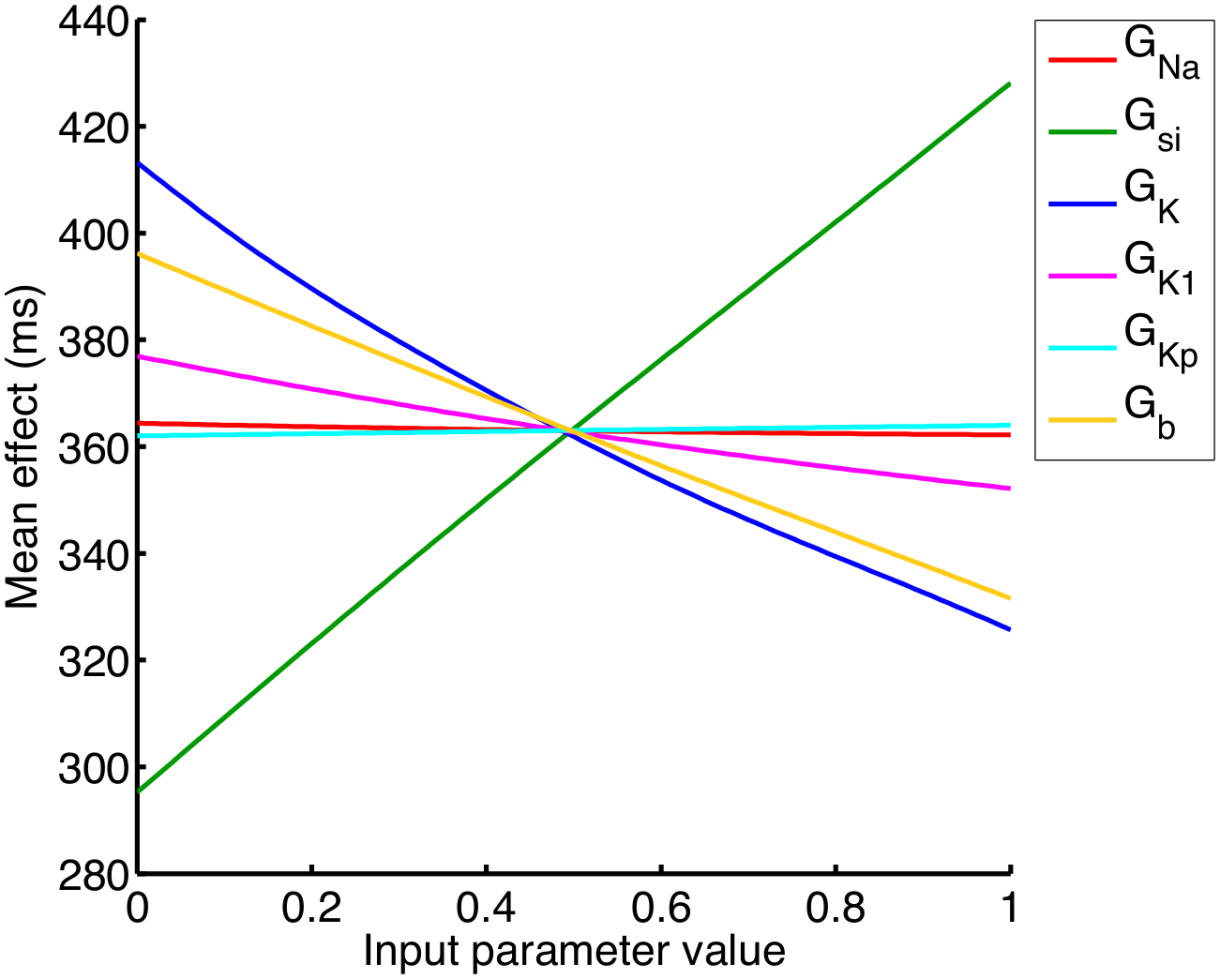
Mean effects in APD_90_ emulator. Mean effect of each of the inputs on APD_90_ as each input is varied whilst the others are held at their mean value.

**Fig 6:**
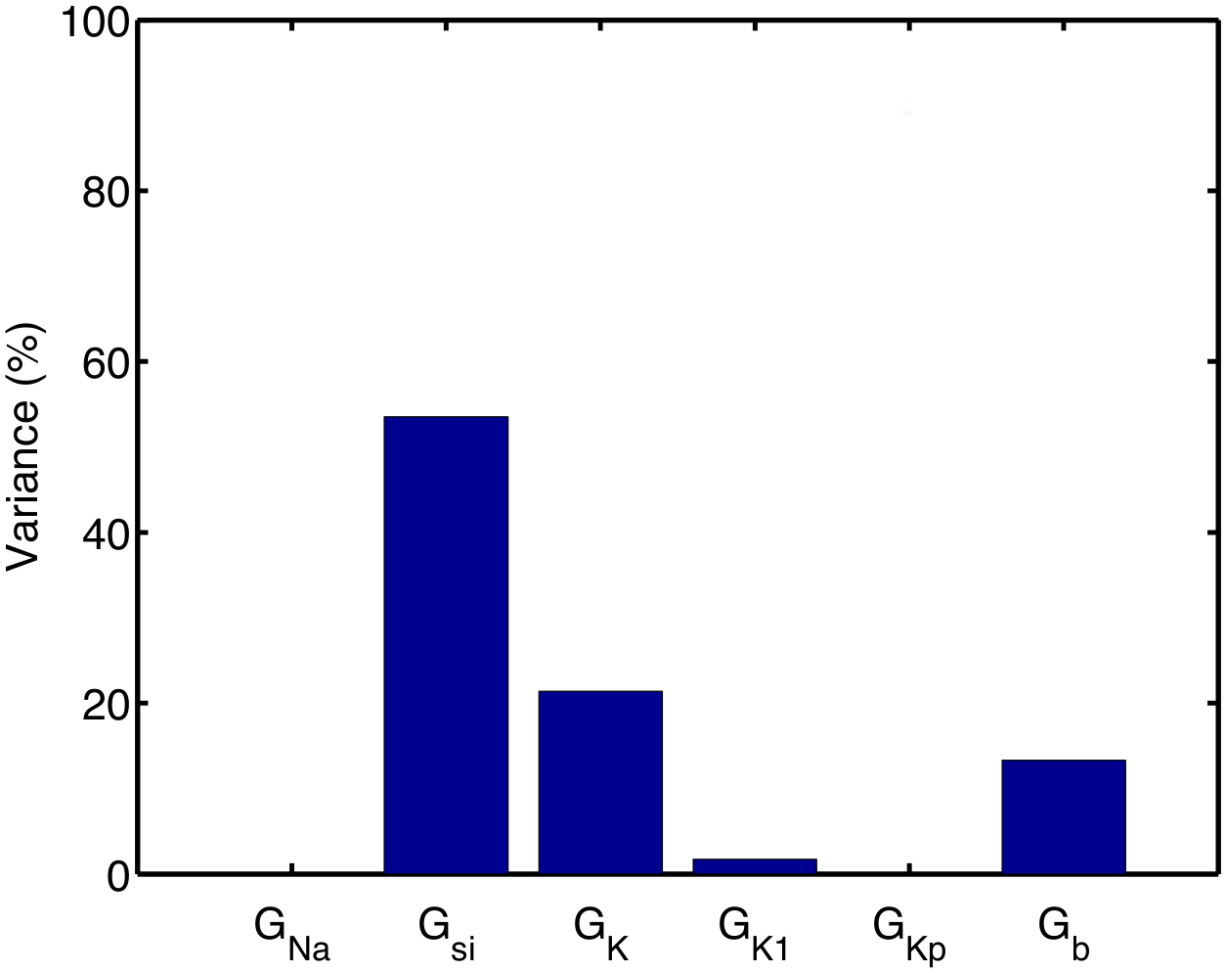
Sensitivity indices in APD_90_ emulator. Sensitivity index of each of the inputs on APD_90_, showing the proportion of total variance that can be attributed to variance in each input.

Figs 5 and 6 show the result of sensitivity analysis using the APD_90_ emulator. Fig 5 shows the mean effects, which are the conditional expectation of the output, as each input in turn was fixed and varied from 0 to 1, while all other inputs were assigned a mean of 0.5 and a variance of 0.04. Increasing G_K_, G_b_, and G_K1_ above their mean value of 0.5 acted to decrease mean APD_90_ in the emulator, whereas increasing G_si_ above 0.5 acted to increase mean APD_90_. These observations are consistent with the operation of the LR1991 model, where G_K_, G_b_, and G_K1_ control outward depolarising currents, and G_si_ controls an inward depolarising current. The trends shown in Fig 5 can also be seen in the scatter plots of the design data in Fig 2.

Fig 6 shows the main effect index (expressed as a percentage) from the APD_90_ emulator for each of the six inputs. We can interpret the main effect index as being the proportional reduction in variance of APD_90_ that would be expected if the input in question was to be fixed. The analysis confirms that G_si_, G_K_, and G_b_ were the most important parameters for determining variability in APD_90_. The sum of these sensitivity indices was 98.4%, indicating that most of the total variance in APD_90_ could be accounted for by the independent effects of variance in the inputs.

### Design data characteristics and evaluation for all emulators

The design data and characteristics of each emulator are given in Table 2, where all inputs were independently normally distributed with a normalised mean of 0.5 and variance of 0.04 (see Table 1 for natural units). In each case, the mean emulator output E*[*E*{*f*(**x**)}] was close to the mean of the simulator output in the design data for each of the emulators, and the variance of the mean emulator output Var*[*E*{*f*(**x**)}] was small, indicating that there little uncertainty was induced in the emulator fit. The coefficient of variation in the emulator output was obtained by dividing the square root of the emulator expectation of the variance E*[*Var*{*f*(**x**)}] by the emulator expectation of the expectation of the simulator output E*[*E*{*f*(**x**)}]. The MD obtained from the initial emulator fit is also given (see S1 Text for details of the MD calculation). In each case, the MD was within ±2 standard deviations of the reference distribution mean, and so the emulators were considered a good fit to the design data.

**Table 2:** Emulator characteristics.

Output	Mean of design data	*E* *[*E*[*f*(x))] of emulator	*Var* *[*E*[*f*(x)]] of emulator	CoV of emulator	Mahanalobis distance
Max. dV_m_/dt (mV/ms)	414.68	415.55	0.000041	6.42%	24.85
Peak V_m_ (mV)	46.67	46.83	0.000006	4.92%	16.06
Dome V_m_ (mV)	10.98	11.00	0.000000	26.87%	14.96
APD_90_ (ms)	365.37	363.02	0.000568	9.48%	28.22
Resting V_m_ (mV)	-84.29	-84.33	0.000000	0.58%	20.64
APD_50_ (ms)	325.77	324.66	0.000733	9.20%	12.89
APDr slope	2.02	2.11	0.000249	22.80%	25.37
Min. DI (ms)	10.02	6.01	0.002152	13.27%	10.58

For each of the outputs, column 2 shows the mean of the design data, obtained from inputs that varied across the normalised range 0..1 given in Table 1. Columns 3-5 show the expectation of emulator output (E*[*E*[*f*(**x**))]), the variance of this expectation Var*[*E*(*f*(**x**))], and coefficient of variation ($\sqrt{E^{*}[Var(f(\text{x}))]}/E^{*}[E[f(\text{x})]]\times 100$) of each emulator, when all of the inputs were assigned a standardised mean of 0.5, and a standardised variance of 0.04 (i.e. 95% confidence intervals of 0.108–0.892). Column 7 shows the Mahanalobis distance between each fitted emulator and an additional 20 test points; a good fit was indicated by a value falling in a distribution with a mean of 20 and a standard deviation of 6.8.

### Mean effects in all emulators

**Fig 7:**
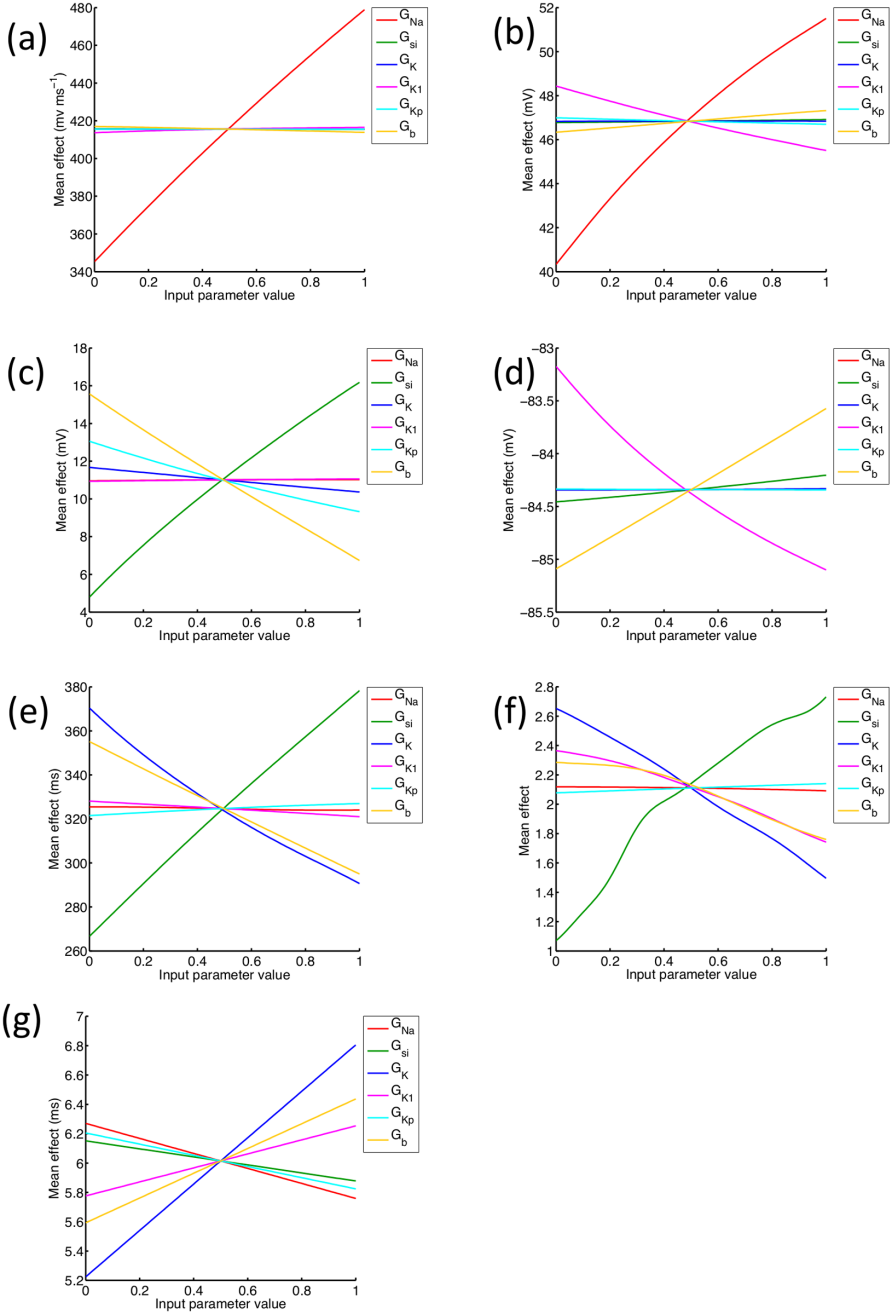
Mean effects in each emulator. Mean effect of each of the inputs on each output as each input is varied whilst the others are held at their mean value.

The mean effects obtained using the emulator for each output are shown in Fig 7. In the LR1991 model the action potential upstroke is controlled by I_Na_, and the Max. dV_m_/dt and Max. V_m_ emulators have captured a strong dependence on G_Na_ as shown in Fig 7(a) and 7(b). Dome voltage (Fig 7(c)) was mainly influenced by G_si_ and G_b_, with increasing G_si_ acting to increase dome voltage, and G_b_ and to a lesser extent G_Kp_ acting in the opposite direction. In the LR1991 model these effects reflect the balance of inward and outward currents during the action potential plateau. Changes in resting voltage (Fig 7(d)) were small, and were controlled by G_b_ and G_K1_, while the mean effects for the APD_50_ emulator (Fig 7(e)) were very similar to those of the APD_90_ emulator (Fig 4), as well as the trends in the design data (Fig 2).

APDr slope is important for the stability of re-entry in cardiac tissue, and the mean effects plot for APDr slope (Fig 7(f)) shows that increasing G_si_ acted to increase APDr slope, while increasing G_K_, G_K1_ and G_b_ acted to decrease the slope. The dependence of APDr slope on G_si_, G_K_, G_K1_ and G_b_ was similar to the dependence of APD, and is also consistent with other studies of re-entry that have used the LR1991 model, where G_si_ has been used to control the slope of the APD restitution curve [32, 33].

### Variance based sensitivity analysis

**Fig 8:**
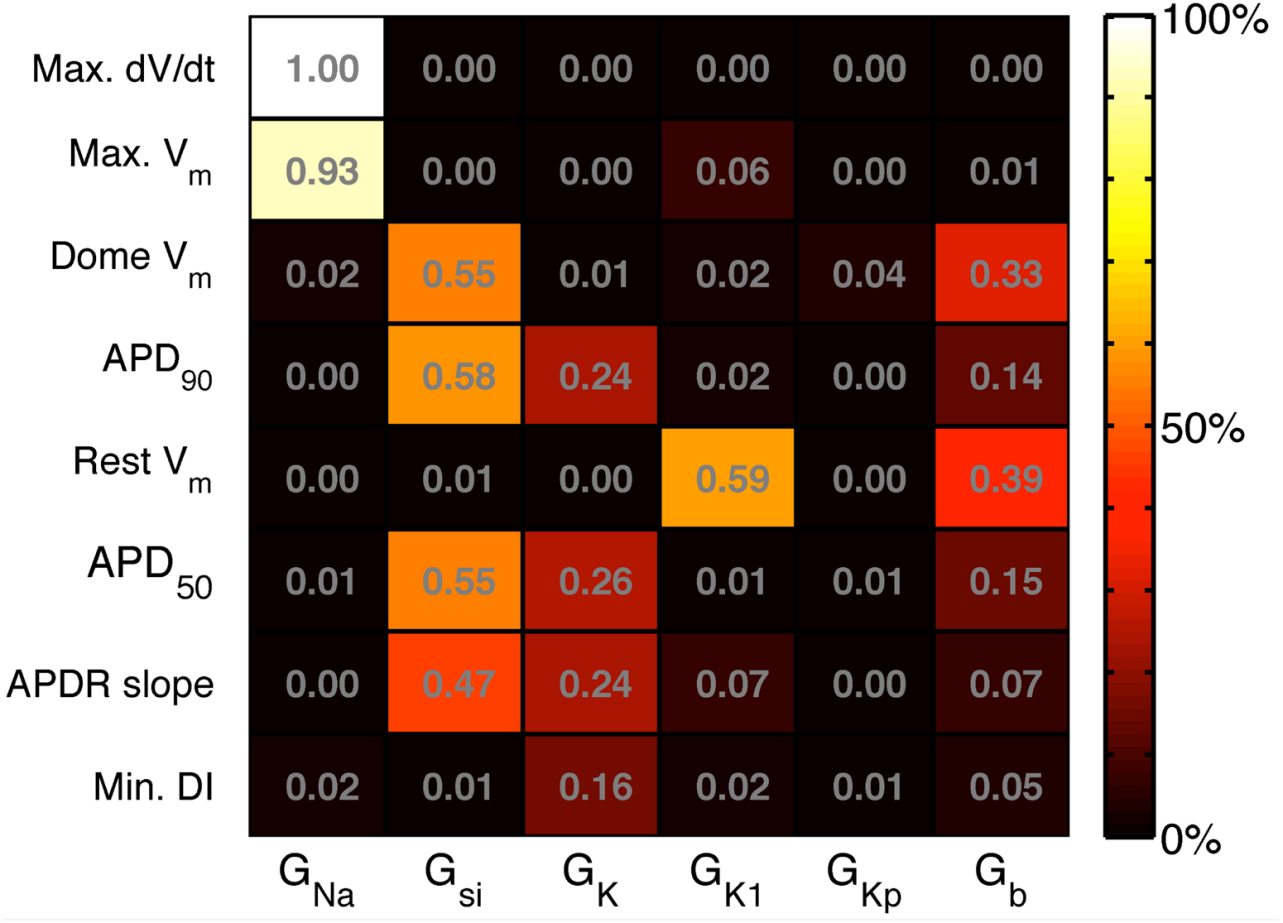
Variance based main effect indices for each emulator. Main effect index of each emulator (rows) to each input (columns), describing the proportion of the output variance that can be accounted for by variance on the input.

Fig 8 shows the main effect indices calculated for each input, and for each emulator. These indices mirror the information shown in the mean effects plots, and indicate, for example, that G_K1_ and G_b_ are the inputs that had the most influence on resting voltage. The sum of the main effect indices for was close to 1 (>98%) for all outputs except the APDr (85%) and the minimum DI (26%). These lower values indicate that 15% of the APDr emulator variance and 74% of the minimum DI emulator variance could be accounted for by interaction effects.

**Fig 9:**
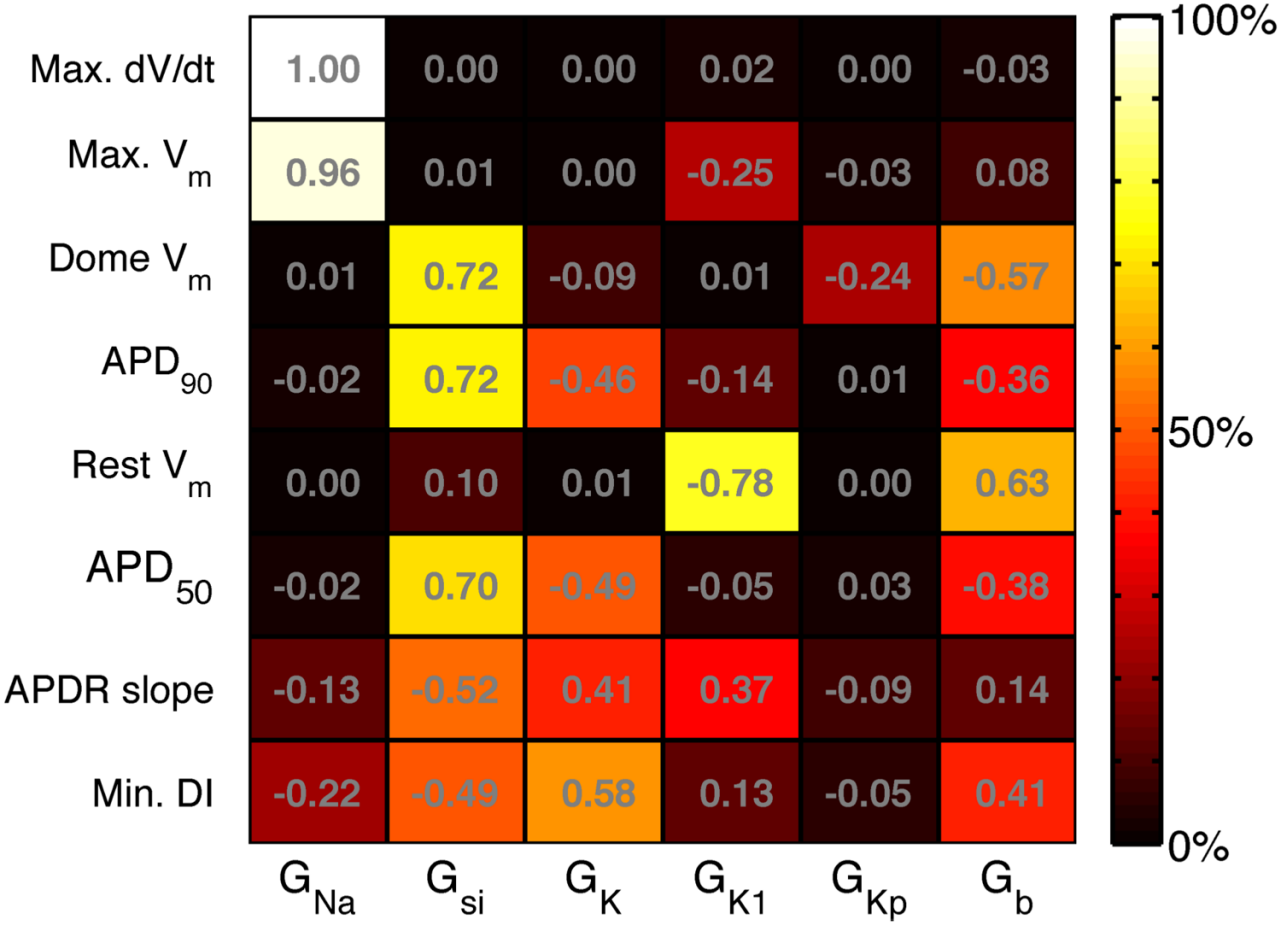
Sensitivity indices calculated with PLS technique. Sensitivity index of each emulator (rows) to each input (columns).

To provide a comparison with the main effects indices obtained from the GP emulators and shown in Fig 8, we also calculated regression coefficients for the LR1991 model using partial least squares (PLS) regression [19]. These coefficients were obtained from the design and test data used to construct the GP emulators, and are shown in Fig 9. These regression coefficients indicate how each output change with each input. Positive values indicate that the output increases as the input increases, whereas negative values indicate that the output decreases as the input increases. The PLS regression coefficients and main effects indices obtained from the GP emulators provide different ways of assessing the way that inputs affect outputs. However the magnitude of the PLS regression coefficients showed good agreement with the main effects indices from the GP emulators. Similar PLS regression coefficients could be obtained using alternative design data drawn from multivariate normal distributions rather than the uniform distribution of the combined design and test data used to construct the GP emulators.

## Discussion

### Summary of findings

In this study we have built a surrogate statistical model of the LR1991 cardiac cell model using, for each model output, a Gaussian process (GP) emulator. The emulators were used to undertake uncertainty and sensitivity analyses of the model efficiently. We found that the GP emulators could be fitted well given a small number of design data points, and we have shown that an emulator approach is a powerful tool for uncertainty and sensitivity analysis in cardiac cell models.

The sensitivity indices shown in Figs 8 and 9 are consistent with what would be expected from our knowledge of the LR1991 model and the underlying physiology that the model represents. They confirm that both GP and PLS emulators have captured the model behaviour correctly. For example, G_Na_ controls the magnitude of the input current during the action potential upstroke, and so would be expected to have a strong influence on maximum dV_m_/dt and peak V_m_. This association is seen in the design data (Fig 2), and the high sensitivity indices in Figs 8 and 9 show that this behaviour has been captured by the emulators. Similarly, G_si_ and G_K_ control the input and output currents during the action potential plateau and repolarisation in the LR1991 model, and so would be expected to influence APD_90_. This association is again seen in the sensitivity indices in Figs 8 and 9.

In the present proof of concept study, we have not attempted to assign uncertainty to the model inputs based on real uncertainties based on experimental errors and variation. However, an important future direction of this research will be to apply an emulator approach to biophysically detailed cell models, where the uncertainty in the mode inputs is based on variability in experimental data.

### Comparison with other approaches

Some of the dependence of outputs on inputs identified with the emulators can be seen in the design data plotted in Fig 2. These data show some of the relationships that are revealed by sensitivity analysis, but other relationships are obscured. For example, although the dependence of Max. dV_m_/dt and Max. V_m_ on G_si_ is clear from Fig 2, the relative influence of G_K_, G_si_ and G_b_ on APD_90_ is hard to quantify from these data alone.

Several recent studies have addressed the problem of parameter sensitivity in cardiac cell models by running large numbers of simulations, where parameters used for each simulation are drawn from a prescribed distribution [12, 17, 18]. Monte Carlo analysis has become one of the standard tools for undertaking sensitivity analysis. However a key advantage of an emulator based approach that is it less computationally demanding. In the present study 220 simulator runs were used to build all of the emulators, and the mean and variance of each emulator output could be calculated directly. In contrast, the Monte Carlo simulations used to generate the comparable data shown in Fig 4(b) required 8000 simulator runs and took several days to compute. Thus an emulator based approach provides a computationally cheaper and more flexible way to examine the sensitivity of a cardiac cell model than ‘brute force’ Monte Carlo.

There are many different ways to build an emulator of a complex model, and other recent studies have used partial least squares (PLS) regression to build emulators that can be used for sensitivity analysis [19, 20]. The sensitivity indices obtained by this approach (Fig 9) identify similar dependencies of outputs on parameters, although the methodology by which the two sets of indices are calculated is different and so there are some quantitative differences. An important benefit of the GP emulator approach over a PLS approach is that uncertainties in inputs are handled explicitly, and so it is possible to examine how the variance of an output changes as an input becomes more uncertain (Fig 4).

### Assumptions, limitations, and difficulties of fitting GP emulators

Although GP emulators are a efficient and powerful tools for uncertainty and sensitivity analysis, the approach described in this study does involve several important assumptions. First, each output is assumed to be a smooth function of the inputs without discontinuities. This assumption was reasonable for most of the emulators examined in the present study, but may underlie problems with the Min. DI emulator and this issue is discussed in more detail below.

Second, it is important that the design data fill the input space evenly. In this study we used a Latin hypercube design to ensure an even distribution of the inputs, and selected the number of design points empirically. We did not undertake a systematic assessment of the minimum number of design points needed to specify each emulator, and this is a topic for future studies with more biophysically detailed cardiac cell models. In addition, other approaches such as orthogonal sampling may provide a more efficient way to cover the parameter space [34].

Third, in this study each GP was described by a linear mean and a Gaussian form for the covariance. As described in S1 Text, this approach enables the direct calculation of integrals involved in the uncertainty and sensitivity analysis. All of the emulators performed well when evaluated with the test data, indicating that our choice of form was satisfactory.

A final assumption was that uncertainty in the emulator inputs and outputs was normally distributed. Experimental data used to construct cardiac cell models are usually assumed to be normally distributed, and are plotted with symmetric error bars [7]. In the present study we did not attempt to estimate the actual uncertainty in inputs because LR1991 is a simplified and generic model. However future studies of more detailed cardiac cell models could assign uncertainties to the inputs that are based on variability in the experimental data used to construct the model, and this would produce valuable insight into the model building process.

While the emulators for most of the outputs behaved as expected, the Min. DI emulator did not. The variance based sensitivity indices shown in Fig 8 are small, and their sum is 0.256. In the design and test data, minimum DI was defined as the shortest DI that could elicit an action potential with APD_90_ longer than 100 ms. It is possible that this constraint leads to a discontinuity in Min. DI in the input space, and careful examination of the design and test data plotted in Fig 2 shows possible evidence of a discontinuity in the dependence of Min. DI on G_si_. However, the sensitivity indices from the PLS emulator shown in Fig 9 were derived from the same design data, and indicate that Min. DI depends mainly on G_si_, G_K_, and G_b_, a tendency also visible in Fig 8 although the absolute values are much smaller. It is possible that Min. DI is influenced by interactions between these inputs, and this would not be accounted for in the sensitivity indices shown in Fig 8.

### Conclusions

In this proof of concept study, we have shown that GP emulators can be used for uncertainty and sensitivity analysis in a model of the cardiac cell action potential. We chose an old and simplified model for this study because its operation is well understood compared to more recent biophysically detailed cell models. We expect that future studies will examine these more detailed models, and will explore the use of GP emulators for uncertainty analysis in multiscale models of cardiac electrophysiology.

## Supporting Information

S1 Text Additional mathematical details. Detailed description of the Gaussian process emulator, how the design data were used to fit the emulator, how the emulator was evaluated, and how it was used for variance based sensitivity analysis.
(PDF)

S1 Data Design data. Design data used to fit the emulators. Data file in comma separated value format with 200 rows, each representing one simulator run. The first 6 columns are the inputs (parameters) in normalised units, and the subsequent columns are the eight outputs.
(CSV)

S2 Data Test data. Test data used to evaluate the emulators. Data file in comma separated value format with 20 rows, each representing one simulator run. The first 6 columns are the inputs (parameters) in normalised units, and the subsequent columns are the eight outputs.
(CSV)
