# Correction: Bayesian Sensitivity Analysis of a Cardiac Cell Model Using a Gaussian Process Emulator

**DOI:** 10.1371/journal.pone.0137004

**Published:** 2015-08-27

**Authors:** Eugene T. Y. Chang, Mark Strong, Richard H. Clayton

There are multiple errors in S1 Text. Please view the correct S1 Text below.

## Supporting Information

S1 Text Additional mathematical details. Detailed description of the Gaussian process emulator, how the design data were used to fit the emulator, how the emulator was evaluated, and how it was used for variance based sensitivity analysis.
(PDF)

## References

[1] Chang ETY, Strong M, Clayton RH (2015) Bayesian Sensitivity Analysis of a Cardiac Cell Model Using a Gaussian Process Emulator. PLoS ONE 10(6): e0130252 doi:10.1371/journal.pone.0130252 26114610 10.1371/journal.pone.0130252 PMC4482712

