# Supplementary material for: Correction: Bayesian Sensitivity Analysis of a Cardiac Cell Model Using a Gaussian Process Emulator
Source: PLoS One. 2015 Aug 27;10(8):e0137004. doi: 10.1371/journal.pone.0137004 (PMC4552007; doi:10.1371/journal.pone.0137004)
Supplement: S1 Text — Detailed description of the Gaussian process emulator, how the design data were used to fit the emulator, how the emulator was evaluated, and how it was used for variance based sensitivity analysis. (PDF) [file pone.0137004.s001.pdf]

Bayesian sensitivity analysis of a cardiac cell model using a Gaussian process emulator – Supporting information

E T Y Chang[1,2], M Strong[3] R H Clayton[1,2,*]

1 Insigneo Institute for in-silico Medicine, University of Sheffield, Sheffield, UK.

2 Department of Computer Science, University of Sheffield, Sheffield, UK.

3 School of Health and Related Research, University of Sheffield, Sheffield, UK.

[*] **E-mail: r.h.clayton@sheffield.ac.uk**

Introduction

In this supporting information, we include details of the mathematics that underpin the approach taken in this study. A much more in-depth coverage of Gaussian process (GP) emulators is given in the MUCM webpages and the toolkit that has been developed there `http://mucm.aston.ac.uk/MUCM/MUCMToolkit/index.php?page=MetaHomePage.html`. Our aim here is to describe the pathway through this material that was followed for the present study. We have used notation that is consistent with the MUCM pages. Vector and matrix quantities are indicated by bold type, and posterior estimates given design data are indicated with an asterisk.

Emulator construction

Each emulator was described by a GP, composed of a mean function and a covariance function,

$$\mathcal{GP}(\mathbf{x}) = h(\mathbf{x})^T\beta + \sigma^2 c(\mathbf{x}, \mathbf{x}'). \tag{1}$$

We used a linear form for the mean function with $h(\mathbf{x}) = (1, \mathbf{x})^T$,

$$h(\mathbf{x})^T\beta = \beta_0 + \beta_1 x_1 + ... + \beta_P x_P, \tag{2}$$

and a Gaussian form for the covariance

$$c(\mathbf{x}_1, \mathbf{x}_2) = \exp\left[-\sum_{p=1}^{P}\left\{\frac{(x_{p,1} - x_{p,2})}{\delta_p}\right\}^2\right]. \tag{3}$$

This choice enabled direct calculation of variance based sensitivity indices. In these expressions $\mathbf{x} = (x_1, x_2, \ldots, x_P)$ are P inputs (parameters), the emulator hyperparameters β and δ are vectors of length P, and σ^2 is a scalar. These quantities were obtained by fitting to the design data, assuming weak prior information on β and σ^2 [2]. In this approach, we first obtained a best estimate for δ, $\hat{\delta}$, from the maximum of the posterior log likelihood given the design data $\mathbf{D}$ and outputs $f(\mathbf{D})$, and and a prior estimate of δ, δ_0. Best estimates of β and σ^2, were then obtained from $\hat{\delta}$.

The value for $\hat{\delta}$ was chosen to be the value that optimised the posterior distribution π^*_δ, given the design data $\mathbf{D}$ and $f(\mathbf{D})$, and assuming a prior distribution of δ,

$$\pi^*_\delta(\delta) \propto (\hat{\sigma}^2)^{-(n-q)/2} |\mathbf{A}|^{-1/2} \, |\mathbf{H}^T \mathbf{A} \mathbf{H}|^{-1/2} \tag{4}$$

Where N was the number of design points (200), $Q = P + 1$, $\mathbf{H}$ an $N \times Q$ matrix given by

$$\mathbf{H} = [h(\mathbf{x}_1), h(\mathbf{x}_2), \ldots, h(\mathbf{x}_N)]^T, \tag{5}$$

$\mathbf{A}$ an $N \times N$ matrix

$$\mathbf{A} = \begin{pmatrix} 1 & c(\mathbf{x}_1, \mathbf{x}_2) & \cdots & c(\mathbf{x}_1, \mathbf{x}_N) \\ c(\mathbf{x}_2, \mathbf{x}_1) & 1 & & \vdots \\ \vdots & & \ddots & \\ c(\mathbf{x}_N, \mathbf{x}_1) & \cdots & & 1 \end{pmatrix}, \tag{6}$$

c the covariance function given by Equation 3, $\mathbf{x}_1, \mathbf{x}_2, \ldots, \mathbf{x}_N$ rows of the design data $\mathbf{D}$, and

$$\hat{\sigma}^2 = \frac{1}{(N - Q - 2)} f(\mathbf{D})^T \left\{ \mathbf{A}^{-1} - \mathbf{A}^{-1}\mathbf{H} \left(\mathbf{H}^T \mathbf{A}^{-1} \mathbf{H} \right)^{-1} \mathbf{H}^T \mathbf{A}^{-1} \right\} f(\mathbf{D}). \tag{7}$$

The value of $\hat{\delta}$ was obtained by maximising equation 4 using the design data as described in the implementation details below. This enabled $\hat{\sigma}^2$ to be calculated from equation 7, and $\hat{\beta}$ was then calculated from

$$\hat{\beta} = \left(\mathbf{H}^T \mathbf{A}^{-1} \mathbf{H} \right)^{-1} \mathbf{H}^T \mathbf{A}^{-1} f(\mathbf{D}), \tag{8}$$

The set of hyper parameters $\hat{\delta}, \hat{\sigma}^2$, and $\hat{\beta}$, together with the mean function given by equation 2 and the covariance function equation 3 specified each emulator.

Emulator validation

Once the emulators were constructed, they could be used to estimate the output (e.g. APD_{90}) for an input $\mathbf{x}$ using the posterior mean and variance of the emulator output given the design data. An asterisk is used to denote a posterior output,. The posterior mean of each emulator output was given by a linear combination of the inputs modified by a term describing the fit to the design data

$$m^*(\mathbf{x}) = h(\mathbf{x})^T\hat{\beta} + c(\mathbf{x})^T\mathbf{A}^{-1}\left(f(\mathbf{D}) - \mathbf{H}\hat{\beta}\right), \tag{9}$$

and the posterior variance by

$$\begin{aligned} v^*(\mathbf{x},\mathbf{x}') = \hat{\sigma}^2\Big\{&c(\mathbf{x},\mathbf{x}') - c(\mathbf{x})^T\mathbf{A}^{-1}c(\mathbf{x}') + \left(h(\mathbf{x})^T - c(\mathbf{x})^T\mathbf{A}^{-1}\mathbf{H}\right) \\ &\left(\mathbf{H}^T\mathbf{A}^{-1}\mathbf{H}\right)^{-1}\left(h(\mathbf{x}')^T - c(\mathbf{x}')^T\mathbf{A}^{-1}\mathbf{H}\right)^T\Big\}. \end{aligned} \tag{10}$$

In these equations the linear mean function $h(\mathbf{x})$ was given by equation 2, the covariance matrix $c(\mathbf{x})$ that depends on $\hat{\delta}$ by equation 3, and the matrices $\mathbf{H}$ and $\mathbf{A}$ by equations 5 and 6 respectively.

These functions were used to validate the emulators against test data $\mathbf{D}^t$ and corresponding outputs $f(\mathbf{D}^t)$ obtained from $N^t = 20$ additional runs of the simulator. For each row $j = 1, \ldots, N^T$ of $\mathbf{D}^t$ with test inputs $\mathbf{x}_j^t$, and test output $y_j^t = f(\mathbf{x}_j^t)$, the standardised error between the predicted emulator output $m^*(\mathbf{x}^t)$ and the observed simulator output y_j^t was calculated from

$$E_j = \frac{y_j^t - m^*(\mathbf{x}_j^t)}{\sqrt{v^*(\mathbf{x}_j^t, \mathbf{x}_j^t)}}. \tag{11}$$

The Mahanalobis distance for the complete set of test data was a measure of overall agreement between the predicted and test data, and was calculated from

$$MD = (f(\mathbf{D}^t) - \mathbf{m}^*)^T\ (\mathbf{V}^*)^{-1}\ (f(\mathbf{D}^t) - \mathbf{m}^*), \tag{12}$$

where $\mathbf{m}^*$ was an $N^t \times 1$ vector $(m^*(\mathbf{x}_1^t), m^*(\mathbf{x}_2^t), \ldots, m^*(\mathbf{x}_{N^t}^t))$, and $\mathbf{V}^*$ is an $N^t \times N^t$ matrix, where each element $\mathbf{V}^*(i,j)$ is $v^*(\mathbf{x}_i^t, \mathbf{x}_j^t)$. The theoretical distribution for MD has a mean of N^t and a variance of $2N^t(N^t + N - Q - 2)/(N - Q - 4)$ [1].

Following validation, a new version of each emulator was built by combining the design data $\{\mathbf{D}, f(\mathbf{D})\}$ and test data $\{\mathbf{D^t}, f(\mathbf{D^t})\}$ and using the $N + N^t = 220$ sets of input and output data to obtain updated values of the emulator hyperparameters $\hat{\delta}$, $\hat{\beta}$ and $\hat{\sigma^2}$. The updated emulator was used for all subsequent analysis.

Uncertainty in emulator output

If the probability density function of uncertain inputs is given by $\omega(\mathbf{x})$, then the posterior expectation of the emulator output (`http://mucm.aston.ac.uk/MUCM/MUCMToolkit/index.php?page=ProcUAGP.html`) with random inputs X is

$$E^*[E[f(X)]] = \int m^*(\mathbf{x})\omega(\mathbf{x})\,dx, \tag{13}$$

and the variance of this expectation was

$$Var^*[E[f(X)]] = \iint v^*(x, x')\omega(x)\omega(x')\,dxdx'. \tag{14}$$

The expected variance of the emulator output was given by

$$E^*[Var[f(X)]] = (I_1 - Var^*[E[f(\mathbf{x}))]) + (I_2 - (E^*[E[f(\mathbf{x}))])^2) \tag{15}$$

where

$$I_1 = \int v^*(x, x)\omega(x)\,dx \tag{16}$$

and

$$I_2 = \int m^*(x)^2\omega(x)\,dx. \tag{17}$$

Our choice of linear mean, weak prior, and Gaussian correlation function for the GP enabled the direct calculation of these integrals, provided that the probability density function of the inputs $\omega(\mathbf{x})$ was multivariate Gaussian with specified mean $\mathbf{m}$ and variance $\mathbf{var}$. The expectation of the emulator output was then

$$E^*[E[f(\mathbf{x}))] = \mathbf{R_h}^T\hat{\beta} + \mathbf{R_t}^T\mathbf{e}, \tag{18}$$

Where $\mathbf{e}$ was an $N \times 1$ vector

$$\mathbf{e} = \mathbf{A}^{-1}(f(\mathbf{D} - \mathbf{H}\hat{\beta}) \tag{19}$$

$\mathbf{R_h}$ a $1 \times Q$ vector

$$\mathbf{R_h} = (1, \mathbf{m}), \tag{20}$$

where $\mathbf{m}$ was a $1 \times P$ vector of the mean values of each input, and $\mathbf{R_t}$ a $1 \times N$ vector, where the kth element (of N) was given by

$$R_t(k) = |\mathbf{B}|^{1/2}\,|2\mathbf{C} + \mathbf{B}|^{-1/2}\,\exp\left(\frac{-Q_k(\mathbf{m}'_k)}{2}\right). \tag{21}$$

In this expression $\mathbf{C}$ was a diagonal $P \times P$ prior correlation matrix where $C(i,j) = \hat{\delta}(i)$ for $i = j$, and $C(i,j) = 0$ otherwise, $\mathbf{B}$ the precision matrix of the input distribution $\omega(\mathbf{x})$, the inverse of a diagonal covariance matrix CV where $CV(i,j) = var(i)$ for $i = j$, and $C(i,j) = 0$ otherwise, and $var(i)$ was the ith element of a $P \times 1$ variance vector $\mathbf{var}$ corresponding to the variance in input i. The function $Q_k(\mathbf{m}'_k)$ yielded a single scalar

$$Q_k(\mathbf{m}'_k) = 2\left(\mathbf{m}'_k - \mathbf{x_k}\right)^T\mathbf{C}\left(\mathbf{m}'_k - \mathbf{x_k}\right) + \left(\mathbf{m}'_k - \mathbf{m}\right)^T\mathbf{B}\left(\mathbf{m}'_k - \mathbf{m}\right) \tag{22}$$

where $\mathbf{m}'_k$ was a $P \times 1$ vector given by

$$\mathbf{m}'_k = \left(2\mathbf{C} + \mathbf{B}\right)^{-1}\,\left(2\mathbf{C}\mathbf{x_k} + \mathbf{B}\mathbf{m}\right), \tag{23}$$

and where $\mathbf{x_k}$ was the kth row of inputs (with P elements) in the design data used to build the emulator.

The variance of the expectation of the emulator output $Var^*[E[f(\mathbf{x}]]$ was given by

$$Var^*[E[f(\mathbf{x})]] = \hat{\sigma}^2\left[U - \mathbf{R}_t^T\mathbf{A}^{-1}\mathbf{R}_t + (\mathbf{R}_h - \mathbf{G}^T\mathbf{R}_t)^T\mathbf{W}(\mathbf{R}_h - \mathbf{G}^T\mathbf{R}_t)\right]. \tag{24}$$

In this expression

$$U = |\mathbf{B}||\mathbf{B2}|^{-1/2} \tag{25}$$

where $\mathbf{B2}$ was a $2P \times 2P$ matrix,

$$\mathbf{B2}(1...P, 1...P) = 2\mathbf{C} + \mathbf{B},$$
$$\mathbf{B2}(P+1...2P, P+1...2P) = 2\mathbf{C} + \mathbf{B},$$
$$\mathbf{B2}(1...P, P+1...2P) = -2\mathbf{C},$$
$$\mathbf{B2}(P+1...2P, 1...P) = -2\mathbf{C}, \tag{26}$$

$\mathbf{G}$ an $N \times Q$ matrix,

$$\mathbf{G} = \mathbf{A}^{-1}\mathbf{H}, \tag{27}$$

and $\mathbf{W}$ a $Q \times Q$ matrix,

$$\mathbf{W} = \mathbf{H}^T\mathbf{A}^{-1}\mathbf{H}. \tag{28}$$

The expectation of the variance of the emulator output $E^*[Var[f(\mathbf{x})]]$ could then be calculated using equation 15, where

$$I_1 = \hat{\sigma}^2\left[1 - trace\left(\mathbf{A}^{-1}\mathbf{R}_{tt}\right) + trace\left(\mathbf{W}\left(\mathbf{R}_{hh} - 2\mathbf{R}_{ht}\mathbf{G} + \mathbf{G}^T\mathbf{R}_t t\mathbf{G}\right)\right)\right] \tag{29}$$

and

$$I_2 = \hat{\beta}^T\mathbf{R}_{hh}\hat{\beta} + 2\hat{\beta}^T\mathbf{R}_{ht}\mathbf{e} + \mathbf{e}^T\mathbf{R}_{tt}\mathbf{e}. \tag{30}$$

In these expressions $\mathbf{R}_{tt}$ was an $N \times N$ matrix, where entry (k, l) was given by

$$R_{tt}(k, l) = |\mathbf{B}|^{1/2}\,|4\mathbf{C} + \mathbf{B}|^{-1/2}\,\exp\left(-Q_{kl}(\mathbf{m'}_{kl})/2\right), \tag{31}$$

where $\mathbf{m'}_{kl}$ was a $P \times 1$ vector

$$\mathbf{m'}_{kl} = (4\mathbf{C} + \mathbf{B})^{-1}\left(2\mathbf{C}x_k + 2\mathbf{C}x_l + \mathbf{B}\mathbf{m}\right), \tag{32}$$

and $Q_{kl}(\mathbf{m'}_{kl})$ a scalar given by

$$Q_{kl}(\mathbf{m'}_{kl}) = 2(\mathbf{m'}_{kl} - x_k)^T\mathbf{C}(\mathbf{m'}_{kl} - x_k) + 2(\mathbf{m'}_{kl} - x_l)^T\mathbf{C}(\mathbf{m'}_{kl} - x_l) \\ +(\mathbf{m'}_{kl} - \mathbf{m})^T\mathbf{B}(\mathbf{m'}_{kl} - \mathbf{m}). \quad (33)$$

$\mathbf{R}_{ht}$ was a $Q \times N$ matrix, where the kth column was given by

$$\mathbf{R}_{ht}(k) = \mathbf{R}_t(k)\mathbf{F}, \quad (34)$$

where $\mathbf{F}$ was a $Q \times 1$ vector with entries $(1, \mathbf{m'}_k(1)...\mathbf{m'}_k(P))$, where $\mathbf{m'}_k$ was given by equation 23. Finally, $\mathbf{R}_{hh}$ was a $Q \times Q$ matrix $\mathbf{R}_h h = \mathbf{R}_h^T\mathbf{R}_h$, where $\mathbf{R}_h$ was given by equation 20.

In the manuscript, Table 2 shows $E^*[E[f(\mathbf{x})]]$, $Var^*[E[f(\mathbf{x})]]$, and $E^*[Var(f(\mathbf{x}))]/E^*[E[f(\mathbf{x})]] \times 100$ for each of the eight emulators, obtained by setting the mean vector $\mathbf{m}$ for the inputs to be $(0.5, 0.5, 0.5, 0.5, 0.5, 0.5)$, and the variance vector $\mathbf{var}$ to be $(0.04, 0.04, 0.04, 0.04, 0.04, 0.04)$. The distributions of APD_{90} in Figure 5(a) were obtained from $E^*[E[f(\mathbf{x})]]$ and $E^*[Var(f(\mathbf{x}))]$ calculated using an identical mean vector with all entries set to 0.5, and a variance vector where all elements were set to 0.0001 except for the element corresponding to G_K, which was set to $0.01, 0.02, 0.05$, and 0.1.

Calculation of mean effects

The mean effect $M_w(x_w)$ shows how the emulator output averaged over uncertain inputs changes when input x_w is given a fixed value. The posterior expectation of $M_w(x_w)$ is a scalar, and is given by

$$M_w(x_w) = \mathbf{R_w}\hat{\beta} + \mathbf{T_w}\mathbf{e}. \quad (35)$$

This equation had the same form as equation 18. $\mathbf{R_w}$ was a $1 \times Q$ vector with $R_w(1) = 1$, and $R_w(i) = x_w$ for $i = w$, and the mean value of input i $m(i)$ otherwise for $i = 2...Q$. $\mathbf{T_w}$ was a $1 \times N$ vector, where the kth element was given by

$$T_w(k) = \left\{ \prod_{i \neq w} \left[\frac{B_{ii}^{1/2}}{(2C_{ii}+B_{ii})^{1/2}} \exp\left(-\frac{1}{2} \left(\frac{2C_{ii}B_{ii}}{2C_{ii}+B_{ii}} \right) (x_{i,k}-m_i)^2 \right) \right] \right\} \times \exp\left(-\frac{1}{2} (x_w - x_{w,k})^T \; 2C_{ww} (x_w - x_{w,k}) \right), \tag{36}$$

where $x_{i/w,k}$ was the value of the i/w^{th} (of P) input on the kth (of N) of the design data used to build the emulator, and all other quantities are defined above.

Calculation of sensitivity indices from the GP emulators

For each emulator, the sensitivity index of each input was calculated from $E^*[V_w]/E^*[Var[f(\mathbf{x})]]$. The total variance in emulator output $E^*[Var[f(\mathbf{x})]]$was calculated as described in equation 15, and the variance in the emulator output when input x_w was fixed $E^*[V_w]$, the sensitivity variance, was calculated from

$$E^*[V_w] = E^*[E[E[f(\mathbf{x}|x_w)]^2]] - E^*[E[f(\mathbf{x})]^2]. \tag{37}$$

The first term in this equation was given by

$$E^*[E[E[f(\mathbf{x}|x_w)]^2]] = \hat{\sigma}^2 \left\{ U_w - trace\left[\mathbf{W} \left(\mathbf{Q}_w - \mathbf{S}_w\mathbf{A}^{-1}\mathbf{H} - \mathbf{H}^T\mathbf{A}^{-1}\mathbf{S}_w^T + \mathbf{H}^T\mathbf{A}^{-1}\mathbf{P}_w\mathbf{A}^{-1}\mathbf{H} \right) \right] \right\} + \mathbf{e}^T\mathbf{P}_w\mathbf{e} + 2\hat{\beta}^T\mathbf{S}_w\mathbf{e} + \hat{\beta}^T\mathbf{Q}_w\hat{\beta}, \tag{38}$$

where U_w was a scalar

$$U_w = \prod_{i \neq w} \left(\frac{B_{ii}}{B_{ii}+4C_{ii}} \right)^{1/2}, \tag{39}$$

P_w was an $N \times N$ matrix, where the $(k,l)^{th}$ element was

$$P_w(k,l) = \left\{ \prod_{i \neq w} \left[\frac{B_{ii}}{2C_{ii} + B_{ii}} \exp\left(-\frac{1}{2} \left(\frac{2C_{ii}B_{ii}}{2C_{ii} + B_{ii}} \right) \left[(x_{i,k} - m_i)^2 + x_{i,l} - m_i)^2 \right] \right) \right] \right\}$$

$$\times \left\{ \left(\frac{B_{ww}}{4C_{ww} + B_{ww}} \right)^{1/2} \exp\left(-\frac{1}{2} \left(\frac{1}{4C_{ww} + B_{ww}} \right) \left[4C_{ww}^2 (x_{w,k} - x_{w,l})^2 + 2C_{ww}B_{ww} \left((x_{w,k} - m_w)^2 + (x_{w,l} - m_w)^2 \right) \right] \right) \right\} \quad (40)$$

Q_w is a $Q \times Q$ matrix, which was assembled in the following steps

$$\begin{aligned}
&Q_w(1,1) = 1 \\
&Q_w(1, 2...Q) = m(1...P) \\
&Q_w(2...Q, 1) = m(1...P) \\
&Q_w(2...Q, 2...Q) = \mathbf{m}\,\mathbf{m}^T \\
&Q_w(w+1, w+1) = Q_w(w+1, w+1) + \mathbf{B}_{ww}^{-1},
\end{aligned} \quad (41)$$

and S_w a $Q \times N$ matrix, where the $(k,l)^{th}$ element was

$$S_w(k,l) = E[h_k(x)] \prod_{1 \leq i \leq P} \left(\frac{B_{ii}^{1/2}}{(2C_{ii}B_{ii})^{1/2}} \right) \exp\left[-\frac{1}{2} \left(\frac{2C_{ii}B_{ii}}{2C_{ii} + B_{ii}} (x_{i,l} - m_i)^2 \right) \right] \quad (42)$$

$$E[h_k(x)] = \begin{cases} 1 & \text{if } k = 1 \\ m_k & \text{if } k \neq w \\ \frac{2C_{kk}x_{k,l} + B_{kk}m_k}{2C_{kk} + B_{kk}} & \text{if } k = w \end{cases} \quad (43)$$

Implementation

All of the code for this study was implemented in Matlab, using expressions detailed in the MUCM toolkit (`http://mucm.aston.ac.uk/MUCM/MUCMToolkit/`). The code was tested against the numerical examples provided in the toolkit.

Each emulator was fitted using design data and outputs obtained from $N = 200$ runs of the LR1991

simulator, so for each emulator $\mathbf{D}$ was an $N \times P$ (200×3) matrix, and $f(\mathbf{D})$ an $N \times 1$ (200×1) vector. The design data were used to construct $\mathbf{H}$. An initial estimate of σ^2 was then obtained from equation 7. Following the MUCM toolkit, we re-parameterised equation 4 with $\tau = 2\, log_e(\delta)$. This removes a constraint on the optimisation because δ ranges from $0 \rightarrow \infty$ whereas τ can range from $-\infty \rightarrow \infty$. Matrix $\mathbf{A}$ was thus calculated from equations 6 and 3 with $\delta = \exp(\tau/2)$, and the Nelder-Mead algorithm as implemented in the Matlab function `fminsearch` was then used to find the minimum of

$$-\pi^*(\tau) = -\pi^*_\delta(\exp(\tau/2)) \tag{44}$$

with a tolerance of 10^{-5} and a maximum number of iterations set to 2000.

A vector of initial values for the correlation length hyperparameters, δ_0, was required for the Nelder-Mead minimisation. We found the fit of the emulator to the test data was sensitive to the choice of δ_0. Good sets of initial estimates (found by trial and error) were $(0.1, 1.0, 1.0, 1.0, 1.0, 1.0)$ for the emulator for maximum dV/dT, $(0.1, 1.0, 1.0, 0.1, 1.0, 1.0)$ for peak V_m, the variance of each column of $\mathbf{D}$ for the emulators for dome voltage, APD_{90}, and resting voltage, and $(1.0, 1.0, 1.0, 1.0, 1.0, 1.0)$ for the remaining emulators.

References

[1] Leonardo S. Bastos and Anthony OHagan. Diagnostics for Gaussian Process Emulators. *Technometrics*, 51(4):425–438, November 2009.

[2] M. C. Kennedy and A O'Hagan. Predicting the output from a complex computer code when fast approximations are available. *Biometrika*, 87:1–13, 2000.
